# AdaBoost Based Multi-Instance Transfer Learning for Predicting Proteome-Wide Interactions between *Salmonella* and Human Proteins

**DOI:** 10.1371/journal.pone.0110488

**Published:** 2014-10-17

**Authors:** Suyu Mei, Hao Zhu

**Affiliations:** 1 Software College, Shenyang Normal University, Shenyang, China; 2 Bioinformatics Section, School of Basic Medical Sciences, Southern Medical University, Guangzhou, China; University of Osnabrueck, Germany

## Abstract

Pathogen-host protein-protein interaction (PPI) plays an important role in revealing the underlying pathogenesis of viruses and bacteria. The need of rapidly mapping proteome-wide pathogen-host interactome opens avenues for and imposes burdens on computational modeling. For *Salmonella typhimurium*, only 62 interactions with human proteins are reported to date, and the computational modeling based on such a small training data is prone to yield model overfitting. In this work, we propose a multi-instance transfer learning method to reconstruct the proteome-wide *Salmonella*-human PPI networks, wherein the training data is augmented by homolog knowledge transfer in the form of independent *homolog instances*. We use AdaBoost instance reweighting to counteract the noise from *homolog instances*, and deliberately design three experimental settings to validate the assumption that the *homolog instances* are effective to address the problems of *data scarcity* and *data unavailability*. The experimental results show that the proposed method outperforms the existing models and some predictions are validated by the findings from recent literature. Lastly, we conduct gene ontology based clustering analysis of the predicted networks to provide insights into the pathogenesis of *Salmonella*.

## Introduction

Pathogen-host protein-protein interaction (PPI) plays an important role in revealing the molecular-level dynamic mechanism of microbial pathogenesis. Fast and accurate reconstruction of proteome-wide pathogen-host PPI networks is essential to reveal the host cellular processes that pathogen proteins may interfere with. In recent years, high throughput experimental techniques have drastically accumulated much knowledge about *intra-specie*s PPI networks, though noisy and far incomplete [1], [2]. Accordingly the majority of computational methods have been developed as the complement of labor-intensive biological experiments for *intra-species* PPI network reconstruction, e.g. yeast PPI network [3], *Arabidopsis thaliana* PPI network [4], human PPI network [5], etc. However, the current host-pathogen PPI networks are comparatively much smaller. The latest HIV-human PPI database [6] contains about 3,638 interactions, the *P.falciparum*-*H.sapiens* PPI dataset [7] contains about 1,112 interactions, and the small *Salmonella*-human PPI data [8] contains only 62 interactions. At present there are very few computational methods developed for pathogen-host PPI networks reconstruction, e.g. HIV-human PPI prediction [9]–[13], *P.falciparum*-*H.sapiens* PPI prediction [14] and *Salmonella*-human PPI prediction [15]–[17]. To improve the predictive performance, most of the reported methods simultaneously leverage a catalog of biological feature information (see Table 1).

**Table 1:** Summary of feature information extracted from literature.

Integration of feature information	Literature
sequence k-mer, interlog, gene ontology, metabolic pathways	[7]
binding motif, gene expression profile, gene ontology, sequence similarity, post-translational modification, tissue distribution, PPI network topology	[9], [10]
protein domain profile, sequence k-mer	[11]
structural similarity	[12]
protein domain profile, gene expression, gene ontology, gene co-expression	[14]

*Salmonella typhimurium* is a facultative intracellular pathogen that causes a variety of diseases from acute gastroenteritis to systemic infection. After invasion into the lumen of host small intestine, *Salmonella* secretes effectors that interact with the host cellular proteins to ensure its survival in the host cellular environment and gain control of the host immune response [18]. To gain more insight into the inflammation/immune signaling pathways that *Salmonella* induced or interfered with, we need to fast and accurately reconstruct the complete *Salmonella*-human PPI networks. Unfortunately, the current experimentally derived *Salmonella*-human PPI network contains only 62 interactions [8], much smaller than the HIV-human PPI network [6] and the *P.falciparum*-*H.sapiens* PPI network [7]. As a fast complement to experimental techniques, computational modeling can accelerate the reconstruction of *Salmonella-*human PPI networks at low cost. To our knowledge, only a few computational methods have been developed to date for *Salmonella*-human PPI prediction [15]–[17]. Schlekera et al. [15] used *protein sequence similarity* and *protein domain similarity* to predict *Salmonella*-human PPIs. Kshirsagar et al. proposed two machine learning methods to predict *Salmonella*-human PPIs [16], [17], wherein the random forest method [16] imputed the missing feature information of *gene ontology* and *gene expression*, and the multi-task learning method [17] proposed DC optimization to integrate the feature information of *gene ontology*, *gene expression* and *pathways*. Except the similarity based method [15], the other two methods both adopt data integration to improve the model performance. Data integration is a popular method to enrich the abundance of feature information, but it has two major disadvantages: (1) aggregating more features without augmenting the size of training data is prone to increase the risk of model overfitting on small data; (2) integration of multiple aspects of feature information poses more demanding data constraints on the computational modeling. If the required feature information is not available for the proteins to be predicted, the data integration methods [8]–[11], [14], [16]–[17] will fail to work. Even for those methods that exploit only one type of feature information, e.g. *protein structural similarity* [12], *gene ontology* [14], etc., the problem of *data unavailability* should also be properly addressed. Most of the types of effective feature information listed in Table 1 are derived from costly experiments and are likely to be not available for some proteins. Thus we need to deliberately consider effective substitution of missing feature information and design proper experimental setting to validate the feature substitution. Kshirsagar et al. [16] conducted explicit substitution for the missing feature information of *gene ontology* and *gene expression*, but did not explicitly estimate the model performance in the case of feature information substitution. As compared to non-sequence feature information, protein sequence is cheap to obtain and imposes the least demanding data constraints on computational modeling. However, it has been reported that protein sequence alone is not sufficient to train a satisfactory model for PPI prediction [19].

In this work, we propose a multi-instance transfer learning method to reconstruct the proteome-wide *Salmonella*-human PPI networks. In the method, *gene ontology* (*GO*) is used as discriminative features to represent proteins. Due to the incompleteness and scarcity of *gene ontology* knowledge, we treat the homolog *GO* information (the aggregated *GO* information from the homologs) as independent *homolog instance* to augment or substitute for the *target instances* (the *GO* information from the protein itself). The potential noise from *homologs* is counteracted by AdaBoost instances reweighting algorithm [20], [21]. To validate the effectiveness of the method, we design the following three experimental settings: (1) *Single Instance Learning* as the baseline model that conducts no homolog knowledge transfer; (2) *Multi-instance Learning Novel* where the training data are represented with *target instances* and *homolog instances*, while the test data are represented with *homolog instances* only; (3) *Multi-instance Learning* where both the training data and the test data are represented with *target instances* and *homolog instances*. Last, we use the proposed method to reconstruct the proteome-wide *Salmonella*-human PPI networks, based on which we further conduct gene ontology based clustering analysis to provide valuable cues for further biomedical research.

## Materials and Methods

### Data and materials

Schleker et al. [8] experimentally derived 62 interactions between 25 *Salmonella* proteins and 51 human proteins, based on which Kshirsagar et al. [16], [17] developed two machine learning methods for *Salmonella*-human PPI prediction. PPI prediction is generally treated as a problem of two-class classification where the PPIs are treated as *positive* data and a *negative* data is needed for computational modeling. At present the experimental *negative* data is hardly available and the common practice to generate *negative* data is random sampling. Random sampling is based on the assumption that the expected number of negatives (non-interacting protein pairs) is several orders of magnitude higher than the number of positives (interacting protein pairs) [22], such that the *negative* space is randomly sampled with larger probability than the *positive* space. The human proteins for negative data sampling are taken from the latest SwissProt database [23]. Besides the way of negative data sampling, the second problem is to determine the ratio of *positive* data to *negative* data. Here we adopt 1∶1 ratio instead of highly skewed ratio like 1∶100 [16], [17] based on the two points: (1) it is hard to simulate the true ratio of *positive* data to *negative* data and simulation of the ratio makes little sense to computational modeling; (2) simply pooling so large a *negative* data is prone to yield an extremely unbalanced training data and thus yields a highly biased model.

To validate how well the proposed model generalizes to unseen data, we further need to construct a validation set from recent literature. We find 18 *Salmonella*-human PPIs in [18] and two novel interactions (SspH2, SGT1) & (SspH2, Nod1) in [24]. After excluding the non-protein interactions (e.g. cholesterol, inositol phospates) and the interactions that have been collected in [8], we obtain 7 novel interactions as validation set.

### Transfer learning

As compared to traditional supervised learning, transfer learning focuses on useful knowledge/information transfer across related domains that are heterogeneously subjected to distinct statistical distributions [25]. One major merit of transfer learning is that there is no need to make the assumption of *independent and identical distribution* (*iid*) between target domain and auxiliary domain. Such the relaxation opens up wide avenues for transfer learning in the field of biological data analysis. In recent years, many sophisticated machine learning methods have been developed to exploit the auxiliary data for useful biological information transfer [26], [27], [28].

In this work, the homolog knowledge is exploited to make up for *data scarcity* as well as to address the concern of *data unavailability*. Unlike computing individual kernel matrices in [26], [27] and training individual classifiers in [28], the homolog knowledge transfer is conducted here by means of independent *homolog instances* under AdaBoost learning framework [20], [21]. The merit is that the independent *homolog instance* is used to augment and enhance the *target instance*, and especially substitute for the *target instance* when the required feature information is not available. Meanwhile the potential of negative knowledge transfer by *homolog instances* can be attenuated by AdaBoost instance reweighting algorithm[20], [21].

### *GO* feature construction

The homologs for each protein are extracted from SwissProt 57.3 database [23] using *PSI-BLast* [29]. We choose *default PSI-BLast* parameters setting (*E-value* = 10) to enlarge the coverage of homologs and *GO* terms. The *GO* terms are extracted from *GOA* database [30] (114 Release, as of 28 November, 2012). For each protein *i*, there are two sets of *GO* terms, one set contains the *GO* terms from homologs denoted as *homolog set* 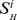, and the other set contains the *GO* terms from the protein itself denoted as *target set* 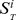. Here the term *target* is used to denote the protein itself (comparative to *homolog*) instead of the pathogen *targeted* protein. Based on the denotations, two feature vectors for each PPI pair (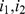) are formally defined as follows:
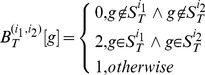
(1)

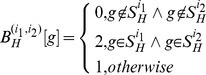
(2)where 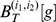 denotes component *g* of the *target instance* 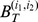 and 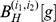 denotes component *g* of the *homolog instance* 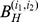. Formula (1) and formula (2) mean that if the interacting proteins pair shares the same *GO* term *g*, the corresponding component in the feature vector 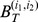
or 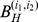
is set 2; if neither protein in the protein pair possesses the *GO* term *g*, the value is set 0; otherwise the value is set 1. The definition is symmetrical, i.e., the protein pair (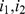) and the protein pair (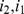) have identical feature representation.

### Multi-instance transfer learning

In the scenario of traditional supervised learning, each data point is represented by only one instance, but only one instance may not be sufficient to depict a complex object in most cases. For example, as biological macromolecules protein molecule constantly changes spatial conformations and DNA molecule temporally changes expression levels. Full depiction of the temporal and spatial information needs more than one instance. For another example, evolutionary information is usually used for us to understand the molecular functions of novel proteins or the interactions between orthologs (*interlog*) [7]. In the case that we can not mingle the information of the protein itself with the information of the homologs, multi-instance representation is a good choice.

In this work, we depict each protein with two instances, the *target instance* and the *homolog instance*, The *target instance* is used to represent the *GO* information of the protein itself and the *homolog instance* is used to capture the evolutionary information of the target protein. Besides enriching the feature information of *target instance*, the *homolog instance* serves the second purpose of substituting the *target instance* when the target *GO* information is not available. We can see that the homolog knowledge transfer by means of independent *homolog instances* is a novel way to simultaneously solve the problems of *data scarcity* and *data unavailability*. Despite the merits, *homolog instances* may carry a certain level of noise from evolutionary divergence and the noise probably does harm to the model performance. For the reason, we need to choose noise-resistant machine learning methods to counteract the noise contained in the *homolog instances*. To our knowledge, AdaBoost is an empirically established and theoretically proven machine learning method that boosts an ensemble of weak learners by instances reweighting [20], [21], [31]. It has been theoretically proven that by means of regularization technique, multiple rounds of instances reweighting help AdaBoost to achieve maximum margin between two-class hyper-planes [31]. The regularization technique penalizes the noise/outlier at the cost of high training error to achieve low generalization error. In this work, we adopt the latest variant Modest AdaBoost [21] that softens the weight distributions between easy-to-classify instances and the hard-to-classify instances. For completeness, Modest AdaBoost [21] is briefly described as follows:

1. Given training instances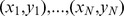, initialize instance weights 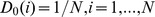;
2. For m  = 1,…, *M* and while 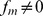

  - Use distribution 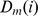
and weighted least squares to train weak classifier:

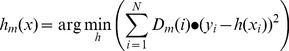
(3)
  - Compute the *inverted* distribution

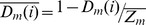
(4)
  - Compute

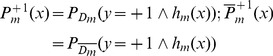
(5)

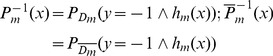
(6)
  - Set

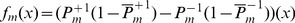
(7)Update the weight distributions
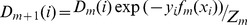
(8)
3. Construct the final classifier

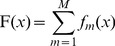
(9)where *M* denotes the number of iterations in the training, 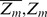
are normalizing coefficients that are chosen to satisfy 
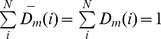
, 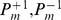
denote the probability that the weak classifier 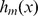 assigns label 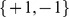
to the instance 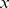
under the weight distribution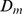, and similarly 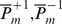
denote the probability under the inverted weight distribution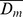. Formula (8) suggests that the weight increases (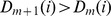) for those misclassified instances (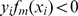). The weight distribution 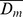 inverted from 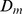
conversely assigns higher weights to those correctly-classified instances. We can see that 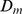
pays more attention to those easy-to-classify instances while 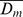
pays more attention to those hard-to-classify instances. Modest AdaBoost made a compromise between the two weight distributions to make soft the decision function 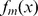 (see formula (7)).

In the test phase, each test protein pair (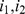) is represented by two instances, the *target instance* 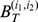
and the *homolog instance* 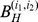. The decision committee 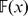 as defined in Formula (9) yields two outputs 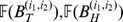 for the two instances (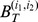,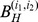). In multi-instance AdaBoost, the final decision value for the protein pair (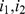) is defined as follows:
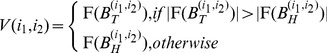
(10)where 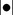
denotes the absolute value. The final class label for the protein pair (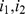) is defined as follows:



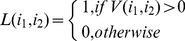
(11)For those positive predictions, the decision values are further normalized to measure the confidence level of prediction:
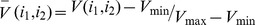
(12)where 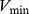
denotes the minimum decision value and 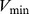
denotes the maximum decision value.

### Model evaluation and model selection

To validate the effectiveness of homolog knowledge transfer by means of independent *homolog instances*, we design three experimental settings: (1) *Single Instance Learning* that does not consider homolog knowledge transfer; (2) *Multi-instance Learning Novel* where the training data point is represented with *target instance* and *homolog instance*, while the test data point is represented with the *homolog instance* only.; (3) *Multi-instance Learning* where both the training data point and the test data point is represented with *target instance* and *homolog instance*. The experimental setting (2) is explicitly designed to estimate the model robustness against *data unavailability*, and the experimental setting (3) is designed to validate the assumption that the *homolog instance* is effective to augment the training data and thus to solve the problem of *data scarcity* for *Salmonella*-human PPI prediction.

As regards with Modest AdaBoost [21], there are two hyper-parameters to be empirically determined, one parameter *M* is the rounds of training, and the other parameter is the base learner. Here *M* is chosen within {50, 100, 150, 200, 250, 300, 350} and the base learner is a decision tree with the number of tree splits chosen within {1, 2, 3, 4, 5, 6, 7, 8, 9, 10}. The model performance is estimated by 10-fold cross validation using the following performance metrics: *ROC-AUC* (*AUC* of *Receiver Operating Characteristic*), *PR-AUC* (*AUC* of *Precision recall curve*), *SP* (*Specificity*), *SE* (*Sensitivity*) and *MCC* (*Matthews correlation coefficient*). We first derive several intermediate variables from confusion matrix *M* as defined in formula (13), and then we calculate *SP*, *SE* and *MCC* for each label (*SP_l_*, *SE_l_* and *MCC_l_*) as defined in formula (14), based on which to further calculate the *overall accuracy* (*Acc*) and the *overall MCC* (*MCC*) as defined in formula (15).
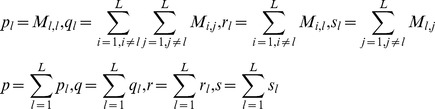
(13)

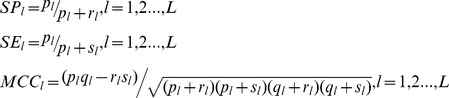
(14)

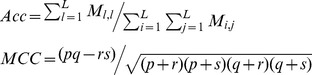
(15)where the confusion matrix 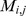
records the counts that class 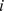 are classified to class 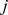 and *L* denotes the number of class labels. The *AUC* metric is calculated on the basis of the decision values defined by formula (10). For comparison with the existing methods, we also report the *F1* score defined as follows:


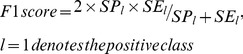
(16)

## Results

### Model performance

#### Cross validation performance evaluation on benchmark data

**Figure 1:**
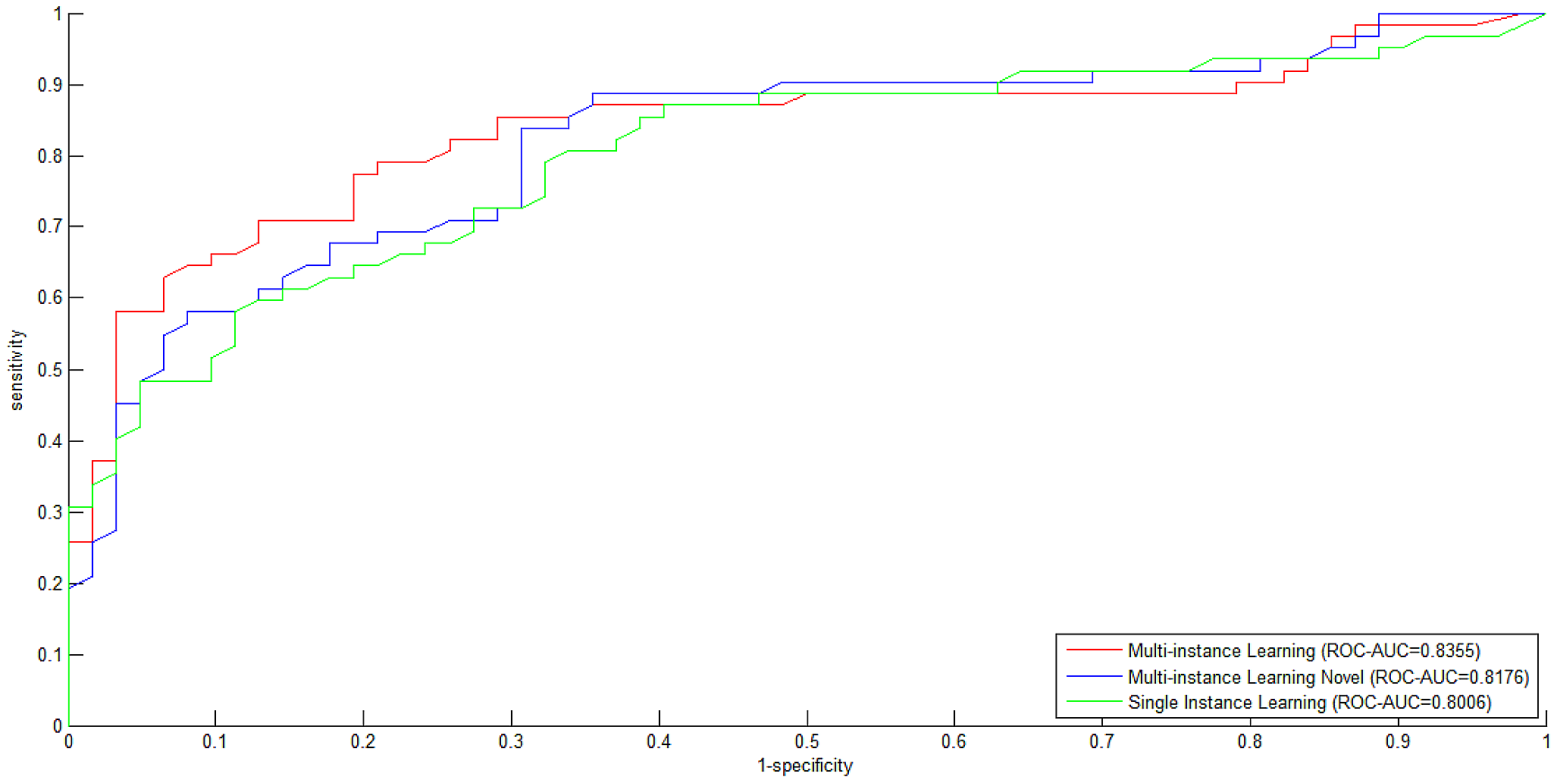
ROC curves for the three experimental settings (Multi-instance Learning, Multi-instance Learning Novel, Single Instance Learning).

**Figure 2:**
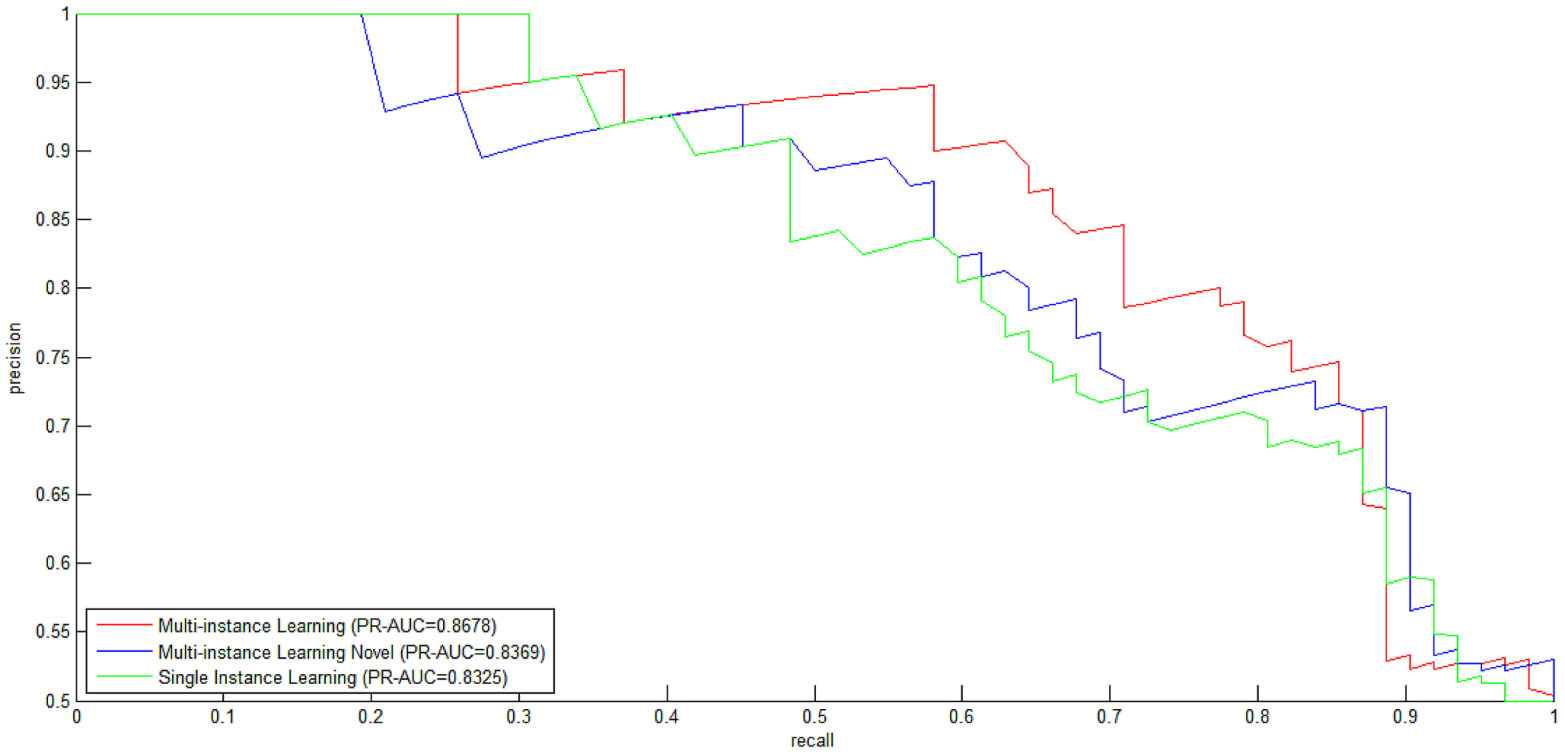
Precision-Recall curves for the three experimental settings (Multi-instance Learning, Multi-instance Learning Novel, Single Instance Learning).

We conduct 10-fold cross validation to validate the effectiveness of homolog knowledge transfer by means of independent *homolog instances*. The *ROC curves* for the three experimental settings are illustrated in Figure 1. From Figure 1, we can see that both the experimental setting *Multi-instance Learning* and the experimental setting *Multi-instance Learning Novel* outperform the baseline experimental setting *Single Instance Learning*, with *ROC-AUC* scores equal to 0.8335, 0.8176 and 0.8003, respectively. Besides *ROC curve*, *precision-recall curve* (*PR curve*) is another performance metric that is often used to measure the performance of two-class classification, especially in the scenario of highly skewed (extremely unbalanced) training data [32]. For comprehensive study, the *PR curves* for the three experimental settings are plot in Figure 2, with *PR-AUC* scores equal to 0.8678, 0.8369 and 0.8325, respectively. From Figure 1 and Figure 2, we can see that the homolog knowledge transfer by means of independent *homolog instances* does improve the model performance (*Multi-instance Learning* versus *Single Instance Learning*), and the *homolog instances* can be treated as effective substitute when the required feature information is not available (*Multi-instance Learning Novel* versus *Single Instance Learning*).

The *ROC Curve* and the *PR Curve* focus on the *positive* class (interaction), paying little attention to the *negative* class (non-interaction). In this work, we comprehensively survey the performance on both he *positive* class and the *negative* class, and use *MCC* to measure the predictive bias. The performance metrics in terms of *SP*, *SE* and *MCC* are given in Table 2. From Table 2, we can see that the *SE*, *SP* and *MCC* values show no significant variance between the *positive* class and the *negative* class in the three experimental settings, except the *SE* values (*positive* class: 0.8065, *negative* class: 0.6935) in the setting *Multi-instance Learning Novel*. The *MCC* values on the two classes suggest that there is little predictive bias. As compared to *ROC-AUC* scores and *PR-AUC* scores, the performance metrics of *overall accuracy* and *overall MCC* shows obvious performance increase between *Multi-instance Learning* and *Single Instance Learning* (*overall accuracy* 78.23% versus 70.97%; *overall MCC* 0.6306 versus 0.5260), so does it between *Multi-instance Learning Novel* and *Single Instance Learning* (*overall accuracy* 75% versus 70.97%; *overall MCC* 0.5833 versus 0.5260). The results once again suggest that the *homolog instances* are effective to augment the training data and substitute for the missing target *GO* information. The very small performance difference between *Multi-instance Learning* and *Multi-instance Learning Novel* further verifies the effectiveness of *homolog instances*.

**Table 2:** Model performance estimation by 10-fold cross validation.

Multi-instance transfer learning		Multi-instance Learning			Multi-instance Learning Novel				Single Instance Learning		
		SP	SE	MCC	SP	SE		MCC	SP	SE	MCC
	**Positive**	0.7692	0.8065	0.6338	0.7246	0.8065		0.5936	0.7031	0.7258	0.5290
	**Negative**	0.7966	0.7581	0.6284	0.7818	0.6935		0.5780	0.7167	0.6935	0.5234
	**Accuracy**	78.23%			75%				70.97%		
	**MCC**	0.6306			0.5833				0.5260		
	**ROC-AUC**	0.8335			0.8176				0.8003		
	**PR-AUC**	0.8678			0.8369				0.8325		
	**F1 Score**	0.80			0.76				0.71		
*Random forest [16]		**SP**					**SE**				
	**Positive**	0.817					0.407				
	**F1 Score**	0.52									
*Multi-task learning [17]	**F1 Score**	0.758									

Note: * denotes the existing models.

The *F1* score defined in formula (16) takes into account *SP* and *SE* on the *positive* class. As shown in Table 2, the three experimental settings *Multi-instance Learning*, *Multi-instance Learning Novel* and *Single Instance Learning* achieve *F1* scores 0.80, 0.76 and 0.71, respectively. The relatively significant increase of *F1* scores also demonstrates the effectiveness of homolog knowledge transfer by means of independent *homolog instances*.

#### Performance comparison with existing models

To further validate the model merits, we compare our proposed multi-instance transfer learning method with two reported machine learning methods, one is Random Forest [16], and the other is multi-task learning method [17]. As shown in Table 3, Random Forest [16] achieved rather low *SE* value (0.407) on the *positive* class, much lower than our method (*SE* = 0.8065), though a little higher *SP* values than our method (*SP* 0.817 versus 0.7692). Such a low *SE* value (0.407) suggests that Random Forest [16] is likely to be highly biased towards the *negative* class. In addition, Random Forest [16] also achieved much lower *F1* score than our method (*F1* score 0.52 versus 0.80).

**Table 3:** Model validation on data from recent literatures.

Salmonella effector proteins	Targeted human proteins	Confidence level
AvrA	MTOR	0.3271
SipA	F-actin caspase-3	0.5157
SipC	F actin	0.5185
PibB2	Kinesin-1	0.2943
SseI	F-actin	0.5185
SspH2	SGT1	0.1059
SspH2	Nod1	0.5410

The multi-task learning method [17] simultaneously exploited multiple PPI networks from the bacteria *S.typhi*, *B.anthracis*, *F.tularensis* and *Y.pestis*. The method reported the *F1* score only (0.758). Model evaluation with inadequate performance metric is one of major weaknesses to both the methods [16], [17]. As compared to the Random Forest method [16] and the multi-task learning method [17], our proposed multi-instance transfer learning method has the following merits (1) we design the experimental setting *Single Instance Learning* as the baseline to verify the performance gain and robustness of information substitution by the *homolog instances*. Unfortunately, the Random Forest method [16] should have designed the baseline experimental setting of no imputing missing values, and the multi-task learning method [17] should have designed the single-task learning of only *S.typhi* without information from *B.anthracis*, *F.tularensis* and *Y.pestis*, as the baseline experimental setting. Without the baseline performance, we can not gain knowledge about the performance increase by imputing missing values [16] and optimizing multiple sub-tasks learning [17]; (2) Besides the baseline experimental setting, we also design an extreme experimental setting *Multi-instance Learning Novel* where the feature information of the test proteins is completely not available (e.g. novel proteins). Both the methods [16], [17] did not test the model robustness against *data unavailability*; (3) Besides *gene ontology*, the two methods [16], [17] also need other feature information such as *gene expression*, *RNAi expression*, *conserved pathways*, *Pfam interactions*, etc., which may also be not available. In our methods, only *gene ontology* is needed and thus the data constraint is much less demanding. Of course, the above-mentioned comparison is *rough* in a sense. The *randomness* introduced by data partition of cross validation and negative data sampling makes it hard to conduct strictly fair comparison between different computational methods. Furthermore, the performance increase of novel method against the existing methods benefits to a certain degree from the update of data (e.g. GOA update). Nevertheless, a relatively rough comparison helps us to gain some knowledge about the reliability of novel models. For the existing models, it is necessary to frequently update the model training using up-to-date data.

Schlekera et al. [15] used protein sequence similarity (sequence identity was 21%) to predict *interlogs*, achieving 49% recognition rate (i.e. *SE* = 0.49) on the dataset [8]. The *SE* value is equivalent to the Random Forest [16] (*SE* = 0.407) but much lower than the proposed multi-instance transfer learning method (*Multi-instance Learning SE = *0.8065; *Multi-instance Learning Novel SE = *0.8065; *Single Instance Learning SE = *0.7258).

#### Model validation on data from recent literature

To further validate the model performance, we conduct independent test using the data from recent literature. The recent experimental findings are generally scarce and scattered in massive biomedical literature, and it is hard to manually collect adequate data for model validation. By manual search, we find 18 *Salmonella-*human PPIs in [18]. Excluding the non-protein interactions (e.g. cholesterol, inositol phospates) and the interactions that have been collected in [8], we obtain 5 novel interactions, together with two novel interactions (SspH2, SGT1) & (SspH2, Nod1) [24], we obtain 7 novel interactions as validation set. The predictions on the validation set are shown in Table 3, where the normalized decision values in the third column measure the confidence level of the predictions. All the 7 novel PPIs are correctly recognized by the proposed method. Interestingly, the two proteins O43765 and O95905 both are related to the gene S*GT1*, but only O43765 is predicted to interact with SspH2. According to *gene ontology* annotations, O95905 is annotated as “*Novel regulator of p53 stability and function*” (http://www.uniprot.org/uniprot/O95905) and O43765 is annotated as “*Co-chaperone that binds directly to HSC70 and HSP70 and regulates their ATPase activity*” (http://www.uniprot.org/uniprot/O43765). It has been reported in [24] that SGT1 interacting with SspH2 was *restricted* to those SGT1 proteins that have NLR *co-chaperone* function. O43765 fulfils *co-chaperone* function while O95905 does not, O43765 (S*GT1*) is predicted to interact with SspH2 while O95905 is not. The details of the predicted interactions between SspH2 and SGT1 are consistent with the experimental findings in [24].

### Reconstruction of proteome-wide *Salmonella*-human PPI networks

#### Proteome-wide PPI predictions

To reconstruct the proteome-wide *Salmonella*-human PPI networks, the 25 *Salmonella* proteins in the 62 interactions [8] are used as pathogen proteins and the human proteins are taken from the file *uniprot_sprot_human.dat.gz* available at ftp://ftp.uniprot.org/pub/databases/uniprot/current_release/knowledgebase/taxonomic_divisions/. Excluding those uncurated proteins and those proteins that already appear in the 62 interactions [8], we totally obtain 20,334 reviewed human proteins. Hence the prediction space contains 508,350 (25×20,334) protein pairs. The predictions are given in File S1. Among the 20,334 human proteins, 6271 human proteins are predicted to interact with the 25 *Salmonella* proteins and 75,381 novel interactions are detected by our method. Comparatively, the Random Forest method [16] predicted 190,868 novel interactions between 22,653 human proteins and 486 *Salmonella* proteins, of which 461 *Salmonella* proteins were not included in the 62 PPIs [8]. After excluding the 461 *Salmonella* proteins and the related predictions, the Random Forest method [16] predicted 134,339 interactions, much larger than 75,381 interactions predicted by our method, suggesting a larger coverage of true interactions and meanwhile a higher risk of false positive predictions.

#### Overlap with existing models

The overlap of predictions between different computational methods is generally small. For instance, the overlap between the predictions by the three methods [9], [12], [13] contains only 4 HIV-human PPIs. Such a low overlap largely results from the two points: (1) the overlap analysis is not based on proteome-wide predictions; (2) the threshold of the final predictions is generally chosen to be much higher than the threshold for model estimation [10], [16], [17], and thus narrows down the scale of the predicted PPI networks. As regards with *Salmonella*-human PPI prediction, the Random Forest method [16] yielded 134,339 interactions, our method yields 75,381 interactions and there are 23,159 overlapped predicted PPIs (see File S2). The overlap rate between Random Forest [16] and our method is 12.41%. Here the overlap rate among *K* methods is formally defined as 
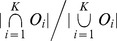
, where 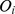 denoted the predictions yielded by the *i*-th method and 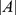 denotes the cardinality of set *A*. The 23,159 overlapped PPIs are supposed to be more reliable. As compared to the low overlap rate among the three methods [9], [12], [13], the large overlap rate between Random Forest [16] and our method is largely due to the proteome-wide prediction space. Besides, as compared to large training data, the small training data is prone to yield a relatively larger portion of false positive predictions, which may also contribute to the large overlap rate. It is worth noting that if we further define as positives those predictions with 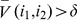
(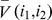 is defined in formula (12)), the predicted interactions are supposed to be more reliable.

Schlekera et al. [15] used the 62 *Salmonella-*human interactions to derive *interlogs*, i.e. the interacting pairs between *Salmonella* protein *orthologs* and human protein *orthologs*. Unlike the Random Forest method [16] and our method, Schlekera et al. [15] did not predict the interactions between the 25 *Salmonella* proteins contained in the benchmark data with other human proteins, so we do not conduct analysis of network overlap with the method.

## Discussions

Reconstruction of pathogen-host PPI networks is of importance to reveal the underlying mechanism of pathogen infection and host defence. At present, it is still a challenging task for labor-intensive experimental techniques to accurately map the proteome-wide pathogen-host protein interactome. Computational modeling is a good complement to experimental methods for fast proteome-wide interactome mapping at low cost. Unfortunately, the current experimentally derived pathogen-host PPI networks are rather small, for instance, there are only 62 known PPIs between *Salmonella typhimurium* and human, such a small network would yield a computational model that does not generalize well. On the other hand, computational modeling need deliberate dwelling on the problem that the knowledge in biological databases (e.g. *gene ontology*) is currently not complete.

In this work, we conduct homolog knowledge transfer by means of independent *homolog instances* to serve the purposes: (1) augmenting the training data to reduce the risk of model overfitting; (2) enriching the feature information to improve the model performance; (3) substituting for the missing feature information to enhance the model robustness against *data unavailability*. The multi-instance transfer learning method is implemented under the framework of AdaBoost, where the *gene ontology* knowledge from the homologs is treated as independent *homolog instance* to augment the training data. The noise from the *homolog instances* could be counteracted by AdaBoost instance reweighting algorithm.

To validate the assumptions that the independent *homolog instances* are effective to solve the problems of *data scarcity* and *data unavailability* for *Salmonella*-human PPI prediction, we deliberately design three experimental settings: *Multi-instance Learning*, *Multi-instance Learning Novel* and *Single Instance Learning* and comprehensively survey the model performance by multiple performance metrics: *SP, SE, Accuracy, MCC, ROC-AUC* and *PR-AUC*. The results of 10-fold cross validation experiments show that the homolog knowledge transfer by means of independent *homolog instances* does improve the model performance and helps the model work properly in the extreme case that the *gene ontology* knowledge is completely not available. The experimental results also show that the proposed multi-instance transfer learning method significantly outperforms the existing machine learning methods with less demanding data constraints. To further validate how well the proposed model generalizes to unseen data, we collect 7 experimentally derived interactions from the recent literatures. Al78 interactions can be correctly recognized by our method. Interestingly, for the two proteins {O43765, O95905} that are related to gene *SGT1*, only the protein O43765 that fulfils the molecular function of *co-chaperone* is predicted to interact with SspH2, which is consistent with the recent findings.

Noteworthily, there are two concerns that need to be addressed, one concern is about the feasibility of training model on the small *Salmonella*-human PPI data, and the other concern is about false positive rate. Although the proposed model demonstrates sound performance from the point of view of cross validation and independent test experiments, the *smallness* of data may not convince us of the validity of the predictions as the large HIV data does [6], [10], [28]. The worry comes from whether or not it is feasible to use so small a data to train a satisfactory model. Actually, some machine learning methods like SVM (*support vector machine*) are derived from small-example statistical learning theory [35], where only a small number of *support vectors* (*SV*) are needed to define the two-class decision hyper-planes and a large number of non-*SV*s are discarded. The gracefulness of *sparseness* is often used to reduce the computational complexity [28], [36], [37]. It has been theoretically proven that the regularization technique helps AdaBoost to achieve large margin between two-class hyperplanes and *sparseness* like SVM [31], implying that it is still feasible to train a satisfactory model on such a small data for *Salmonella*-human PPI prediction. We note that the multi-instance transfer learning method is specifically proposed to augment very small data. For large data like HIV, the proposed method will increase the computational complexity and thus seems not to be a proper solution. As regards with the second concern, both biological experiments and computational predictions are supposed to yield a certain level of false positives. For biological experiments, different experimental techniques are generally specific to particular types of interactions, for instance, Y2H as an *in vivo* technique is highly effective at detecting transient interactions and can be readily applied to screen large genome-wide libraries, but is limited by its biases toward non-specific interactions [38]. Likewise computational method is also prone to yield false positive predictions, especially on small training data, and the 7 correctly recognized interactions (see Table 3) may be worried to result from high false positive rate. Actually, our proposed method greatly reduces the risk of false positive predictions as compared to the Random Forest [16] (predicted 75,381 interactions versus predicted 134,339 interactions). To ensure the validity of the predictions, we can increase the decision threshold as 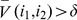
(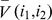
is defined in formula (12)).

The ultimate goal of model development and model estimation is to reconstruct reliable proteome-wide protein interaction networks between *Salmonella typhimurium* and Homo sapiens. To gain insights into the patterns of *Salmonella* infection and host response, the predicted PPI networks are further subjected to *gene ontology* based clustering analysis. We simply cluster together the *Salmonella* targeted human proteins on the basis of *gene ontology* terms. To provide valuable cues about the host protein complexes, molecular functions and signaling pathways that *Salmonella* proteins may interfere with, we assign to the same cluster the human partners that possess the same *GO* term, with each cluster corresponding to one PPI sub-network. All the *GO* terms of human proteins are classified into thee major classes, i.e., biological processes (P), molecular functions (F) and cellular compartments (C). For each major class, we further discuss the two cases: (1) all the 25 *Salmonella* proteins are involved in the PPI sun-network, denoted as P1, F1 and C1, respectively; (2) NOT all the 25 *Salmonella* proteins are involved in the PPI sun-network, denoted as P2, F2 and C2, respectively. P1, F1 and C1 are given in File S3, File S4 and File S5, respectively. P2, F2 and C2 are given in File S6, File S7 and File S8, respectively. For the sake of quite a large number of predicted PPI sub-networks, we only demonstrate several PPI sun-networks as examples, interested readers are referred to File S3∼File S8 for insightful biological cues.

### PPI sub-network GO: 0032862 - activation of Rho GTPase activity

**Figure 3:**
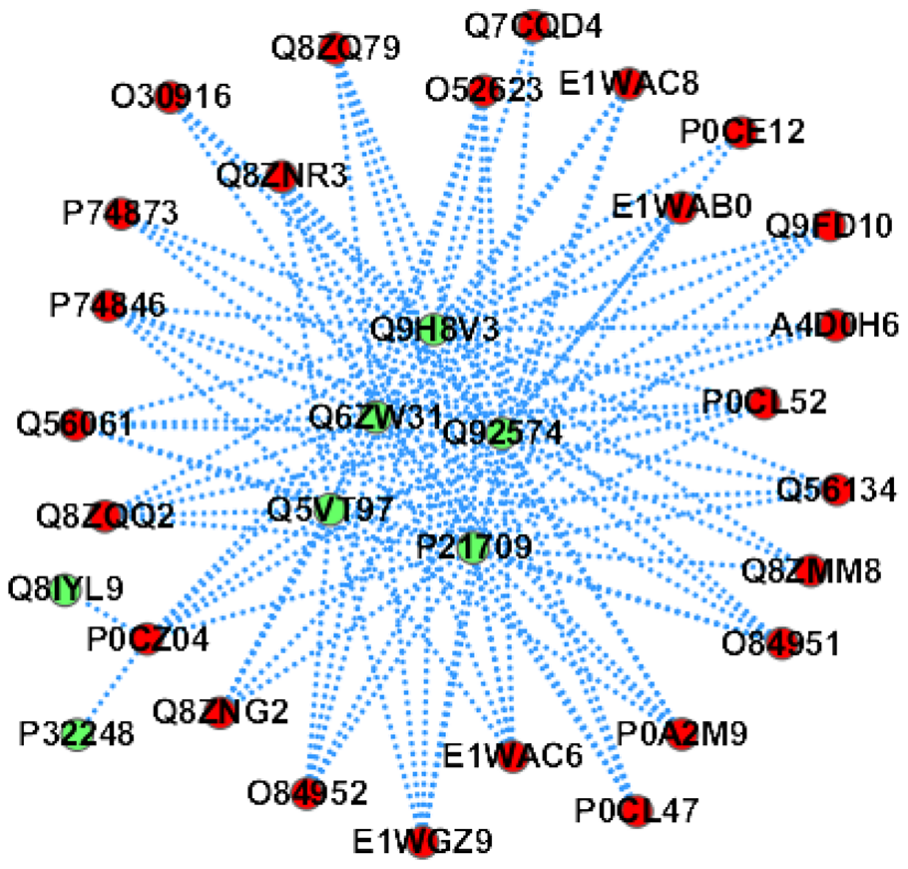
The predicted *Salmonella*-human PPI sub-network GO: 0032862 (*biological process*: *activation of Rho GTPase activity*). The red node denotes *Salmonella* protein and the green node denotes human protein.

It has been reported in [33] that *Salmonella typhimurium* injects toxins SopE, SopE2 and SptP to change the GTP/GDP loading state of Rho GTPases by transient interactions, wherein SopE and SopE2 mimic eukaryotic G-nucleotide exchange factors (GEF) and thereby activate Rho GTPase signaling pathways in the infected host cells. In this work, the *Salmonella* proteins are predicted with 7 human proteins that are annotated with *GO* term GO: 0032862 (activation of Rho GTPase activity). The predicted PPI sub-network GO: 0032862 is illustrated in Figure 3. The two *Salmonella* SopE proteins (O52623, Q7CQD4) are predicted to interact with 5 human proteins {Q9H8V3, Q6ZW31, Q5VT97, Q92574, P21709}, suggesting that the SopE activates Rho GTPase signaling pathways by directly mimicking the molecular functions of the host proteins or indirectly interacting with the host proteins. Among the 7 human proteins, the two human proteins {Q9H8V3, Q92574} are predicted to be targeted by all the 25 *Salmonella* proteins while the two proteins {Q8IYL9, P32248} are predicted to be targeted by only one *Salmonella* protein.

### PPI sub-network GO: 0008234 - cysteine-type peptidase activity

**Figure 4:**
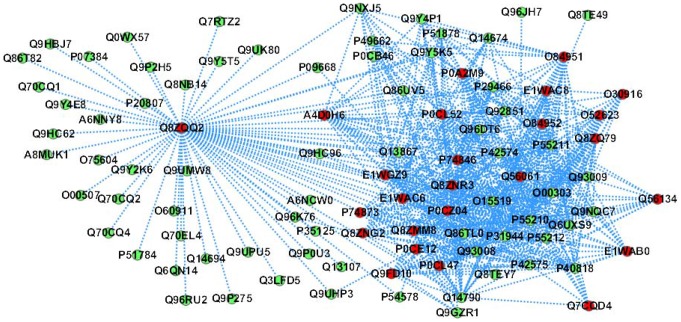
The predicted *Salmonella*-human PPI sub-network GO: 0008234 (*molecular function*: *cysteine-type peptidase activity*). The red node denotes *Salmonella* protein and the green node denotes human protein.

The predicted PPI sub-network is illustrated in Figure 4. In Figure 4, the *Salmonella* proteins are predicted to interact with 70 human proteins that fulfil the molecular function “cysteine-type peptidase activity” (GO: 0008234), wherein the *Salmonella* protein Q8ZQQ2 is a hub protein that is predicted to target 63 human proteins. The *Salmonella* protein slrP (Q8ZQQ2) is an effector protein that functions to alter the host cell physiology and promote bacterial survival in the host tissues. This protein is an E3 ubiquitin ligase that interferes with the host ubiquitination pathway and leads to significant decrease of thioredoxin activity and increase of the host cell death (http://www.uniprot.org/uniprot/Q8ZQQ2). The six human proteins {P5521, Q14790, P42575, O00303, P55210, P55211} are predicted to be targeted by all the 25 *Salmonella* proteins, wherein P5521 is involved in the activation cascade of caspases responsible for apoptosis execution, whose over-expression promotes programmed cell death. The predictions suggest the *Salmonella* proteins may also interfere with the host apoptotic signaling pathways.

### PPI sub-network GO: 0007205 - activation of protein kinase C activity by G-protein coupled receptor protein signaling pathway

**Figure 5:**
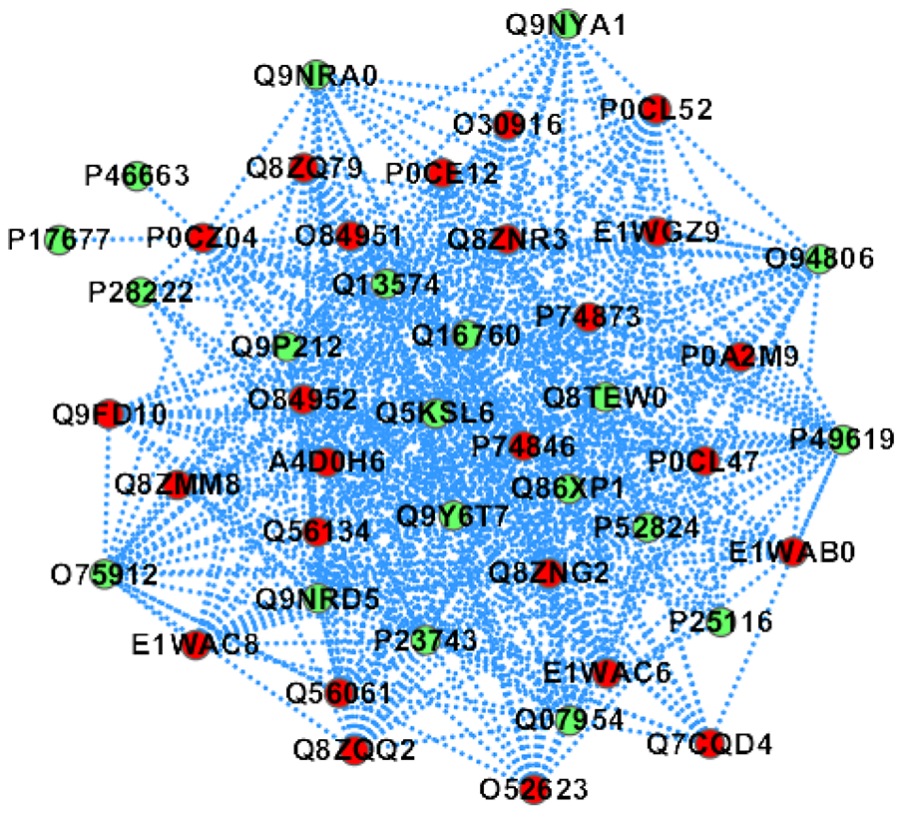
The predicted Salmonella-human PPI sub-network GO: 0007205 (biological process: activation of protein kinase C activity by G-protein coupled receptor protein signaling pathway). The red node denotes Salmonella protein and the green node denotes human protein.

The predicted PPI sub-network is illustrated in Figure 5. As shown in Figure 5, all the 20 predicted human partners except {P46663, P17677} are densely connected with the 25 *Salmonella* proteins. *Salmonella* protein spiC (P0CZ04) is predicted to target all the 20 human proteins and the human proteins {Q07954, P49619, Q86XP1, Q9NRD5, Q16760, P52824, Q9Y6T7, Q9P212, Q8TEW0, Q5KSL6, P23743} are predicted to be targeted by all the 25 *Salmonella* proteins. *Salmonella* protein spiC (P0CZ04) is a virulence protein that plays a central role in mammalian macrophage infection by inhibiting phagosome-lysosome fusion and cellular trafficking (http://www.uniprot.org/uniprot/P0CZ04). The human protein Q07954 is an endocytic receptor involved in endocytosis and in phagocytosis of apoptotic cells, and may modulate the cellular events such as APP metabolism, kinase-dependent intracellular signaling, neuronal calcium signaling as well as neurotransmission (http://www.uniprot.org/uniprot/Q07954). The molecular function annotations of the two proteins suggest that *Salmonella* protein spiC (P0CZ04) is highly likely to interact with the human protein Q07954.

### PPI sub-network GO: 0003777 - microtubule motor activity

**Figure 6:**
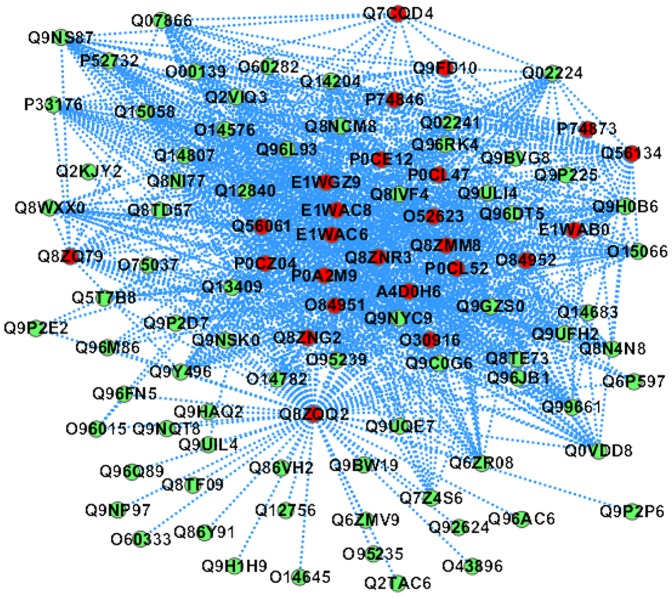
The predicted *Salmonella*-human PPI sub-network GO: 0003777 (*molecular function*: *microtubule motor activity*). The red node denotes *Salmonella* protein and the green node denotes human protein.

In [34], it is reported that *Salmonella typhimurium* grows within the host cells in a permissive compartment termed *Salmonella*-containing vacuole (SCV), and some SPI-2 effectors modulate microtubule motor activity on the SCV. In this work, the *Salmonella* proteins are predicted with 74 human proteins that are annotated with the *GO* term GO: 0003777 (microtubule motor activity) (see Figure 6), and the SPI-2 effector sseJ (Q9FD10) is predicted to interact with 26 human proteins that are involved in the molecular functions “microtubule motor activity”. From Figure 6, we can see the predicted PPI sub-network GO: 0003777 is densely connected and the 10 human proteins {Q9H0B6, Q8NCM8, Q8NCM8, Q9GZS0, Q12840, P33176, Q02224, Q9NS87, O95239, O14576} are predicted to be targeted by all the 25 *Salmonella* proteins. The human protein Q9H0B6 (Kinesin light chain 2) is a light chain of Kinesin (a microtubule-associated force-producing protein that may play a role in organelle transport), and the light chain may function in coupling of cargo to the heavy chain or in the modulation of its ATPase activity (http://www.uniprot.org/uniprot/Q9H0B6). The prediction suggests that the *Salmonella* proteins may interfere with the host organelle transport and ATPase activity.

## Supporting Information

File S1 XML file contains the proteome-wide predicted *Salmonella*-human PPIs.
(XML)

File S2 XML file contains the overlapped interactions between our method and Random Forest [16].
(XML)

File S3 XML file contains the clusters of interacting human partners that participate in specific *biological processes*. All the 25 *Salmonella* proteins are involved in the predicted PPI sub-networks.
(XML)

File S4 XML file contains the clusters of interacting human partners that fulfil specific *molecular functions*. All the 25 *Salmonella* proteins are involved in the predicted PPI sub-networks.
(XML)

File S5 Text file contains the clusters of interacting human partners that are localized in specific *cellular compartments*. All the 25 *Salmonella* proteins are involved in the predicted PPI sub-networks.
(XML)

File S6 XML file contains the clusters of interacting human partners that participate in specific *biological processes*. NOT all the 25 *Salmonella* proteins are involved in the predicted PPI sub-networks.
(XML)

File S7 XML file contains the clusters of interacting human partners that fulfil specific *molecular functions*. NOT all the 25 *Salmonella* proteins are involved in the predicted PPI sub-networks.
(XML)

File S8 XML file contains the clusters of interacting human partners that are localized in specific *cellular compartments*. NOT all the 25 *Salmonella* proteins are involved in the predicted PPI sub-networks.
(XML)
